# *In silico* Identification of Serovar-Specific Genes for *Salmonella* Serotyping

**DOI:** 10.3389/fmicb.2019.00835

**Published:** 2019-04-24

**Authors:** Xiaomei Zhang, Michael Payne, Ruiting Lan

**Affiliations:** School of Biotechnology and Biomolecular Sciences, The University of New South Wales, Sydney, NSW, Australia

**Keywords:** *Salmonella enterica*, accessory genomes, serotyping, serovar-specific gene markers, lineage-specific gene markers, polyphyletic serovars, paraphyletic serovar, serovar prediction

## Abstract

*Salmonella enterica* subspecies *enterica* is a highly diverse subspecies with more than 1500 serovars and the ability to distinguish serovars within this group is vital for surveillance. With the development of whole-genome sequencing technology, serovar prediction by traditional serotyping is being replaced by molecular serotyping. Existing *in silico* serovar prediction approaches utilize surface antigen encoding genes, core genome MLST and serovar-specific gene markers or DNA fragments for serotyping. However, these serovar-specific gene markers or DNA fragments only distinguished a small number of serovars. In this study, we compared 2258 *Salmonella* accessory genomes to identify 414 candidate serovar-specific or lineage-specific gene markers for 106 serovars which includes 24 polyphyletic serovars and the paraphyletic serovar Enteritidis. A combination of several lineage-specific gene markers can be used for the clear identification of the polyphyletic serovars and the paraphyletic serovar. We designed and evaluated an *in silico* serovar prediction approach by screening 1089 genomes representing 106 serovars against a set of 131 serovar-specific gene markers. The presence or absence of one or more serovar-specific gene markers was used to predict the serovar of an isolate from genomic data. We show that serovar-specific gene markers have comparable accuracy to other *in silico* serotyping methods with 84.8% of isolates assigned to the correct serovar with no false positives (FP) and false negatives (FN) and 10.5% of isolates assigned to a small subset of serovars containing the correct serovar with varied FP. Combined, 95.3% of genomes were correctly assigned to a serovar. This approach would be useful as diagnosis moves to culture-independent and metagenomic methods as well as providing a third alternative to confirm other genome-based analyses. The identification of a set of gene markers may also be useful in the development of more cost-effective molecular assays designed to detect specific gene markers of the all major serovars in a region. These assays would be useful in serotyping isolates where cultures are no longer obtained and traditional serotyping is therefore impossible.

## Introduction

*Salmonella* causes human salmonellosis and infections of warm-blooded animals (Kingsley and Bäumler, 2000). The *Salmonella* genus is divided into two species, *S. enterica* and *S. bongori*. serotyping further classifies *Salmonella* into over 2,600 serotypes (serovars) through the agglutination reaction of antisera to three surface antigens O, H1, and H2 (Le Minor and Bockemühl, 1984; Le Minor et al., 1990). There are 46 O antigens, that identify the serogroup. Together with 119 H1 and H2 flagellin antigens, the O, H1, and H2 combinations identify the serovars. Only a small proportion of the serovars are responsible for the majority of the human *Salmonella* infections (Popoff et al., 2004).

Serotyping by antigenic agglutination is being replaced by molecular serotyping (Cai et al., 2005; Wattiau et al., 2011). This can be achieved through examination of the sequence of O antigen gene cluster, H1 antigen encoding gene *fliC* and H2 antigen encoding gene *fljB* (Fitzgerald et al., 2007). O antigen gene clusters can be differentiated by presence or absence of genes while H1 and H2 antigens are differentiated by sequence variation (McQuiston et al., 2004; Guo et al., 2013; Zhang et al., 2015). *Salmonella* serotypes may also be inferred through MLST (Wattiau et al., 2011; Achtman et al., 2012) as a serotype may be inferred by its sequence types. However, a prerequisite for this approach is that prior knowledge of the corresponding relationship of serovar to sequence type is required.

Recently, with the development of whole-genome sequence-based comparison, several studies have identified genomic markers as an alternative molecular method for serotyping. Zou et al. (2016) identified seven genes that provide sufficient resolution to differentiate 309 *Salmonella* strains representing 26 serovars and found serovar-specific genes in 13 out of 26 serovars. Laing et al. (2017) identified genomic fragments specific to *Salmonella* species and subspecies through pan-genome analysis. These specific genes or DNA fragments have been used as molecular targets to develop multiple molecular assays for rapid identification and detection of *Salmonella* at species and serovar level. However, these specific genes or DNA fragments are limited in their discriminative ability due to their ability to only distinguish a smaller number of serovars.

In this study, we aimed to use the extensive publicly available collection of *Salmonella* genomes to identify serovar-specific gene markers for the most frequent *Salmonella* serovars. We show the potential of these serovar-specific gene markers as markers for molecular serotyping either *in silico* typing of genomic data or for development of laboratory diagnostic methods.

## Materials and Methods

### Ribosomal MLST ST Based Isolate Selection

The *Salmonella* database in the Enterobase (Alikhan et al., 2018) as of March 2018 was queried and 118997 isolate were examined. Representative isolates for each rSTs were selected and extracted by an in-house python script. Only serovars with more than four rSTs were included in this study. For the 20 largest serovars representative isolates were only randomly selected from rSTs with two or more isolates. For the remaining serovars, one representative isolate for each rST was randomly selected. Raw reads for these isolates were retrieved from ENA (European Nucleotide Archive^1^) and were *de novo* assembled using SPAdes v3.10.1 assembler with default settings^2^ (Bankevich et al., 2012). The serovar of the assembled genomes was predicted by SISTR (Yoshida et al., 2016) after they met the following criteria which were defined by Robertson et al. (2018) using QUAST^3^ (Gurevich et al., 2013): assembly size between 4 and 6 Mb with the number of contigs less than 500, the largest contig greater than 100 kb, GC content between 50 and 54%, gene predicted by glimmer within QUAST more than 3000. The concordance between the resulting SISTR serovar predictions and the reported serovar on the Enterobase metadata record were examined and a small number of genomes were removed from analysis due to inconsistent serovar predictions. The final data set consisted of 2258 high quality genomes with consistent serovar prediction representing 107 serovars (Supplementary Table S1).

### Identification of *Salmonella* Serovar-Specific Candidate Gene Markers

To determine the potential serovar-specific gene markers for 107 serovars, the 2258 genomes were annotated using PROKKA (Seemann, 2014). Pan-genome and core-genome were analyzed by roary (Page et al., 2015) using an 80% sequence identity threshold. The genes specific to each serovar were identified from the pan-genome’s accessory genes with an in-house python script. In this study, the number of genomes from a given serovar containing a specific gene for that serovar was termed true positive (TP), the number of genomes from the same serovar lacking the same gene was termed false negative (FN). The number of genomes from other serovars containing the same serovar-specific gene was termed false positve (FP). Relaxed cutoffs (20% FN, 10% FP) were used initially in order to ensure that all serovars had candidate specific genes which could be further investigated. Paralogous genes were removed from the analyses.

### Evaluation of Potential Serovar- Specific Gene Markers

The F_1_ score was used for initial selection of the potential serovar-specific gene markers. F_1_ score was evaluated based on the formula: 2 × (PPV × Sensitivity)/(PPV + Sensitivity), where PPV was defined as TP/(TP+FP) and sensitivity [true positive rate (TPR)] was defined as TP/(TP+FN). The F_1_ ranges from 0 to 1, where 1 means the serovar-specific gene which was present in all genomes of a given serovar and absent in all genomes of other serovars. The serovar-specific gene markers were selected using the best performing gene for each serovar based on F1 score. The specificity [true negative rate (TNR)] defined as TN/(TN+FP) was used to evaluate true negative (TN) rate of serovar-specific gene markers. False positive rate (FPR) was defined by 1 – TNR.

### Phylogenetic Analyses

In order to determine the causes for the observed false negative and FPRs in the candidate serovar-specific gene markers, the phylogenetic relationships of the serovars involved were investigated. The draft assemblies of 1258 isolates were used to generate phylogenetic trees by using parsnp v1.2^4^ (Treangen et al., 2014) with default parameters to determine the phylogeny between and within serovars. The tree was visualized by FigTree v1.4.3 (Schneider et al., 2000).

### Location and Functions of Serovar-Specific Gene Markers

Representative complete genomes for each serovar containing gene features were downloaded from NCBI^5^ and were used to determine the location of each of candidate serovar-specific gene by BLASTN with default settings (version 2.2.6, Supplementary Table S2). In serovars with no representative complete genome a representative genome was selected from isolates assembled in this study. Sequences of serovar-specific gene markers are included in Supplementary Data S1. Clustering of genes across the genome was used to investigate whether the serovar-specific gene markers were potentially part of a single element gained by a serovar in one event. The candidate serovar-specific gene markers were considered as a cluster if they were located less than 5 kb from each other.

The functional categories of gene markers were identified from RAST annotation^6^ (Aziz et al., 2008). The prophage sequences within serovars reference genomes were identified by using PHASTER to indicate whether the serovar-specific gene markers may have been acquired along with prophages (PHAge Search Tool Enhanced Release) (Arndt et al., 2016).

### *In silico* Serotype Prediction Using Serovar-Specific Gene Markers

An additional 1089 isolates were selected from the Enterobase using an in-house python script with the exclusion of 2258 isolates used for the initial screening from the same database as of March 2018 (Supplementary Table S3). BLASTN was used to search against the 1089 genomes belonging to 106 *Salmonella* serovars for the presence of any of the serovar-specific gene markers. Custom python scripts were then used to predict serovar from these serovar assignments based on the known gene presence pattern for each serovar. The TP was classified as the total number of correctly assigned serovars and cases where the correct serovar was called as well as one or more FP. Failed assignment was defined where no serovar or incorrect serovars were called. Serovar predictions were compared to SeqSero (Zhang et al., 2015) and SISTR predictions.

### Calculation of the Specificity of Candidate Serovar-Specific Gene Markers for Common Serovars

The specificity of typing rate for common serovars (Hendriksen et al., 2011) was equal to (1 – potential error rate). The potential error rate of serovar-specific gene markers defined by the formula: (Number of FPs)^∗^(The frequency of that serovar in a given region)/(Total of genomes of that serovar).

## Results

### Identification of Candidate Serovar-Specific Gene Markers

**FIGURE 1:**
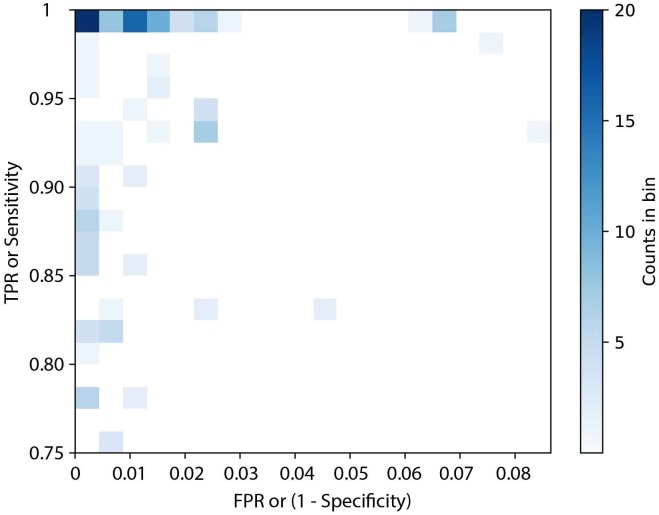
The distribution of sensitivity and specificity of 354 potential serovar-specific gene markers. TPR, true positive rate; FPR, false positive rate. Where a gradient from light blue (low percentage) to dark blue (high percentage) is displayed.

The accessory genes from 2258 genomes representing 107 serovars were screened to identify potential serovar-specific gene markers. This initial screening identified 354 potential serovar-specific gene markers within 101 serovars. Six serovars namely, Bareilly, Bovismorbificans, Thompson, Reading, Typhi, and Saintpaul had no candidate serovar-specific gene markers that were present in all lineages of a given serovar. The specificity (TNR) and sensitivity (TPR) of the 354 candidate serovar-specific gene markers were also examined and summarized in Figure 1. Forty serovars contained 194 serovar-specific gene markers with 100% specificity and sensitivity (no FN or FP), while 31 serovars contained 80 candidate serovar-specific gene markers with 100% sensitivity but with less than 100% specificity (varied FP). Nine serovars contained 27 candidate serovar-specific gene markers with 100% specificity but with less than 100% sensitivity (varied FN). The remaining 21 serovars contained 53 candidate serovar-specific gene markers with both specificity and sensitivity less than 100% (varied FN and FP).

We constructed a phylogenetic tree using 1258 representative isolates from 107 serovars using ParSNP (Supplementary Figure S1). The 1258 isolates were selected based on phylogenetic relationships of the initial 2258 isolates from which we selected isolates to represent each independent lineage. We found that members of each of the 82 serovars formed a monophyletic lineage while 24 serovars were polyphyletic with each made up of 2 to 4 lineages. Several of these serovars are known to be polyphyletic and are unlikely to contain serovar-specific gene markers (Falush et al., 2006; den Bakker et al., 2011; Achtman et al., 2012; Timme et al., 2013). Serovar Enteritidis is paraphyletic with three other serovars (Dublin, Berta, and Gallinarium) arising from within the larger Enteritidis clade which is itself made up of three lineages known as clade A, B and C (Graham et al., 2018). The five Enteritidis-specific candidate gene markers were negative to the Enteritidis isolates which clustered separately on the tree.

Interestingly for four polyphyletic serovars, Bredeney, Kottbus, Livingstone and Virchow, each had one candidate serovar-specific gene which was present in all isolates of that serovar. For the remaining 20 polyphyletic serovars and paraphyletic serovar Enteritidis, we searched for lineage-specific gene markers as each serovar contained more than one lineage. If all lineages contained at least one lineage-specific gene, we regard that serovar as containing serovar-specific gene markers. A total of 111 potential lineage-specific gene markers were identified for 19 polyphyletic serovars and paraphyletic serovar Enteritidis, among which, 27 lineage-specific gene markers were identified for 5 serovars with 100% specificity and sensitivity (no FN and FP), 76 candidate lineage-specific gene markers for 14 serovars with 100% sensitivity and less than 100% specificity (varied FP), and Enteritidis containing 6 candidate lineage-specific gene markers with varied FN and FP (Table 1).

**Table 1:** Lineage-specific candidate gene markers for polyphyletic serovars and paraphyletic serovar.

	No of	No of		No of
Serovar	genomes	lineages	Lineages	genes	Sensitivity^#^	Specificity^#^
Bareilly	20	2	Bareilly-I	2	100.00	98.76
			Bareilly-II	1	100.00	99.11
Bovismorbificans	34	2	Bovismorbificans-I	1	100.00	97.25
			Bovismorbificans-II	1	100.00	99.91
Bredeney	5	2	Bredeney	1	100.00	97.61
Cerro	40	2	Cerro-I	4	100.00	100.00
			Cerro-II	2	100.00	100.00
Derby	24	3	Derby-I&II	1	100.00	100.00
			Derby-III	4	100.00	100.00
Enteritidis	165	2	Enteritidis-clade A/C	1	100.00	98.85
			Enteritidis-clade B	5	96.43*	99.65
Give	26	3	Give-I&II	4	100.00	94.60
			Give-III	1	100.00	99.82
Havana	20	2	Havana-I	2	100.00	97.39
			Havana-II	4	100.00	100.00
Hvittingfoss	16	3	Hvittingfoss-I&II	1	100.00	100.00
			Hvittingfoss-III	1	100.00	100.00
Kentucky	31	2	Kentucky-I	5	100.00	100.00
			Kentucky-II	3	100.00	100.00
Kottbus	12	3	Kottbus	1	100.00	93.98
Livingstone	17	2	Livingstone	1	88.24*	99.47
London	11	2	London-I	2	100.00	99.11
			London-II	3	100.00	99.87
Mississippi	14	2	Mississippi-I	5	100.00	100.00
			Mississippi-II	1	100.00	100.00
Newport	85	3	Newport-I&II	1	100.00	92.87
			Newport-I&III	1	100.00	91.67
Oranienburg	29	4	Oranienburg-I&II&IV	1	100.00	98.67
			Oranienburg-III	1	100.00	98.72
Oslo	9	2	Oslo-I	2	100.00	99.91
			Oslo-II	1	100.00	100.00
Paratyphi B	72	3	Paratyphi B-I&II	11	100.00	97.83
			Paratyphi B-III	1	100.00	100.00
			Paratyphi B-mono	1	100.00	100.00
Reading	8	2	Reading-I	1	100.00	100.00
			Reading-II	2	100.00	99.96
Saintpaul	31	3	Saintpaul-I	11	100.00	98.14
			Saintpaul-II	5	100.00	100.00
			Saintpaul-III	1	100.00	98.27
Senftenberg	27	3	Senftenberg-I&II	2	100.00	99.96
			Senftenberg-III	1	100.00	100.00
Stanleyville	6	3	Stanleyville-I&II	2	83.33*	95.44
Tell El Kebir	8	2	Tell El Kebir-I	3	100.00	100.00
			Tell El Kebir-II	6	100.00	100.00
Thompson	32	2	Thompson-I	2	100.00	98.49
			Thompson-II	2	100.00	100.00
Virchow	39	2	Virchow	1	100.00	100.00


*^∗^The sensitivity of less than 100% was due to at least one target serovar genome lacking the candidate gene. Six out of 165 isolates of Enteritidis, two out of 17 isolates of Livingstone-I and one out of 6 isolates of Stanleyville-III lacked candidate lineage-specific gene markers. ^#^Sensitivity and specificity for the best performing gene for each lineage. The number of isolates used to arrive at Sensitivity and Specificity calculation for each serovar-specific gene marker were listed in Supplementary Table S2*.

For the 11 of the 82 monophyletic serovars that lacked serovar-specific candidate gene markers due to FN, we found that the FN was often due to isolates that are grouped on one branch and diverged earlier from the other isolates. For such groups, we searched for lineage-specific gene markers. Therefore, two or more gene markers can be used to identify a serovar and such serovars were also considered to contain serovar-specific gene markers, similar to polyphyletic serovars. Three serovars, Paratyphi A, Heidelberg, and Muenchen could be identified by the combined lineage-specific gene markers.

A total of 414 candidate serovar-specific gene markers including 295 serovar-specific gene markers and 119 lineage-specific gene markers are summarized in Supplementary Table S2. In total, 106 of 107 serovars contained one or more gene markers, 33 serovars contained one specific gene while 73 contained two or more gene markers. There were no candidate serovar-specific gene markers found for monophyletic Typhi and no potential lineage-specific gene markers found for lineage III of Stanleyville which contained only one isolate.

### Functional Categories of Serovar-Specific Gene Markers

Functional characterization of all 414 gene markers identified for the 106 serovars using RAST found that 197 had known functions and 217 encoded hypothetical proteins with unknown functions. Only 46 genes with annotations can be grouped into functional categories while 151 genes with functions were not in RAST functional categories (Table 2). Using PHASTER. 45 candidate serovar-specific gene markers were located within predicted prophages.

**Table 2:** Serovar-specific genes functional categories.

Category by RAST	No of genes^∗^
DNA Metabolism	18
Regulation and cell signaling	5
Carbohydrates	2
Membrane Transport	8
Virulence, Disease and Defence	1
RNA Metabolism	4
Stress Response	2
Cofactors, Vitamins, Prosthetic Groups, Pigments	1
Cell Wall and Capsule	1
Phages related	2
Protein Metabolism	1
Amino Acids and Derivatives	1
Uncategorized	152
Hypothetical proteins with unknown function	217


*^∗^The details of these genes were listed in Supplementary Table S2*.

### A Minimal Set of Serovar-Specific Gene Markers for *in silico* Molecular Serotyping

**FIGURE 2:**
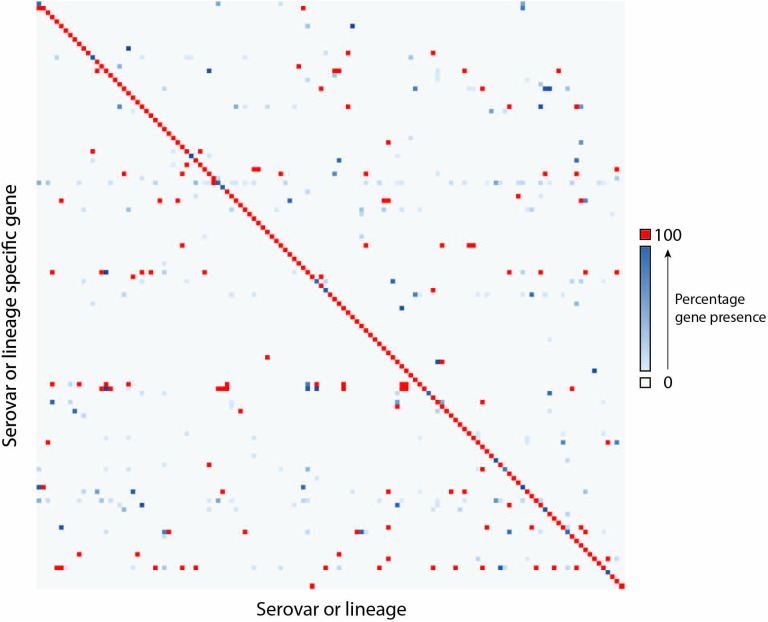
The distribution of a minimal set of 131 serovar-specific genes in 106 serovars. The Y-axis shows serovar or lineage-specific gene markers and the X-axis shows serovars or lineages. The details were listed in Supplementary Table S4. Gray indicated zero genomes containing a gene (TN). Gene/Genome pairs along the diagonal represent genomes containing the serovar-specific gene markers that matches their serovar (TP). Red represents genes that are present in 100% of genomes for a given serovar or lineage. Where a gene is present in less than 100% of a serovar a gradient from light blue (low percentage) to dark blue (high percentage) is displayed. Blue pairs along the diagonal represent the presence of FN. Pairs that are blue or red outside of the diagonal represent pairs containing genes that do not match the predicted serovar of the genome (FP).

For many serovars, multiple candidate serovar-specific gene markers or lineage-specific gene markers were identified. In these cases, a single gene was selected that has the lowest FN and FP rates. A minimum of 131 gene markers allows identification of the serovars with error rates from 0 to 8.33%. The distribution of the gene markers across all 106 serovars demonstrates high degree of specificity as shown in Figure 2 in which the diagonal displays the one to one relationship of the serovar or lineage with serovar-specific gene markers while the off-diagonal space showed sparse scattered presence of these genes in other serovars of varied percentages indicating a low FPR. The details of these gene markers were listed in Supplementary Table S4. Overall, 45 serovars can be distinguished by their respective serovar-specific gene and 61 serovars can be differentiated by a combination of gene markers.

We tested an additional 1089 genomes belonging to 106 non-typhoidal *Salmonella* serovars to evaluate the ability of the 131 specific gene markers to correctly assign serovars to isolates. Using the serovar-specific gene markers, 1038 of the 1089 isolates (95.3%) were successfully assigned [924 to correct serovar with no FP or FN (84.8%) and 114 to the correct serovar with some FP (10.5%)] and 51 failed (4.7%). For SISTR and SeqSero, the number of concordant serovar assignments were 1037 (95%) and 905 (82.8%), respectively (Supplementary Table S3).

### Serovar-Specific Gene Markers for Serotyping of Common Serovars

The top 20 serovars causing human infection found in each continent (Hendriksen et al., 2011) were collapsed into a combined list of 46 serovars (Supplementary Table S5). Since these serovars contained the vast majority of isolates causing human infections globally, we consider them separately to assess the utility of candidate serovar-specific gene markers for serotyping of most prevalent serovars in a local setting. When only these serovars were considered, 18 out of 46 could be uniquely identified by one of the serovar-specific gene markers. To increase accuracy of typing in the remaining 28 common serovars where serovar-specific gene markers have varied FPRs, we examined using subsets of the 131 gene markers (ranging from 2 to 9 genes per serovar) to eliminate potential FP. For example, the combination of Choleraesuis specific gene and Cerro-I lineage-specific gene can eliminate false positive isolate of Cerro from Choleraesuis, if both genes are positive, the isolate could be assigned Cerro while if Cerro-I lineage-specific gene is negative, the isolate is Choleraesuis.

To estimate potential errors in typing, we took into account the frequency of the 46 common serovars that showed large differences between regions (Hendriksen et al., 2011). Therefore, different combinations of genes may be used to specifically limit false positive results from serovars present in that region. In a given region, the specificity of common candidate serovar-specific gene markers was calculated using the rate of FP and the frequency of the false positive serovar in that region. The specificity of candidate serovar-specific gene markers was also calculated using the FP rate (Supplementary Table S4). For example, a panel of 15 genes could be used for typing the 10 most frequent serovars in Australia (NEPSS 2010) (Table 3). When Australian regional frequencies were taken into account, the genes listed in Table 3 can be used as markers for laboratory based typing and the error rate will be less than 2.4%.

**Table 3:** A panel of serovar-specific genes for typing the ten most frequent serovars in Australia.

Serovar	Gene 1	Gene 2	Gene 3	Gene 4	Gene 5	Gene 6	Gene 7	Gene 8	Gene 9	Gene 10	Gene 11	Gene 12	Gene 13	Gene 14	Gene 15
Typhimurium	+	-	-	-	-	-	-	-	-	-	-	-	-	-	-
Enteritidis-B	-	+	-	-	-	-	-	-	-	-	-	-	-	-	-
Enteritidis-A/C	-	-	+	-	-	-	-	-	-	-	-	-	-	-	-
Virchow	-	-	-	+	-	-	-	-	-	-	-	-	-	-	-
Saintpaul-I	-	-	-	-	+	-	-	-	[+]	-	-	-	-	-	-
Saintpaul-II	-	-	-	-	-	+	-	-	-	-	-	-	-	-	-
Saintpaul-III	[+]	-	-	-	-	-	+	-	-	-	-	-	-	-	-
Infantis	-	-	-	-	-	-	-	+	-	-	-	-	-	-	-
Paratyphi B-I&II	-	-	-	-	-	-	-	-	+	-	-	-	-	-	-
Paratyphi B-III	[+]	-	-	-	-	-	-	-	-	+	-	-	-	-	-
Chester	-	-	-	-	-	-	-	-	-	-	+	-	-	-	-
Hvittingfoss-I&II	-	-	-	-	-	-	-	-	-	-	-	+	-	-	-
Hvittingfoss-III	[+]	-	-	-	-	-	-	-	-	-	-	-	+	-	-
Muenchen-I	-	-	-	-	-	-	[+]	-	-	-	-	-	-	+	-
Muenchen-II	-	-	-	-	-	-	-	-	-	-	-	-	-	-	+
Error rate	2.4	0	1.5	0	2.9	0	0.2	0	1	0	2.2	0	0	0	0.9
Specificity	97.6	100	98.5	100	97.1	100	99.8	100	99	100	97.8	100	100	100	99.1


*“+”: true positives (TP); “-”: true negatives (TN); [+]: false positives (FP) in a subset of genomes. Gene 1 = STM4494 (Typhimurium); Gene 2 = SEN1384 (Enteritidis-clade B); Gene 3 = R561_RS18155 (Enteritidis-clade A/C); Gene 4 = SEV_RS01820 (Virchow); Gene 5 = SESPA_RS08460 (Saintpaul-I); Gene 6 = SeSPB_A1749 (Saintpaul-II); Gene 7 = Saintpaul-III; Gene 8 = L287_RS37190 (Infantis); Gene 9 = SPAB_01124 (Paratyphi B-I&II); Gene 10 = SPAB_01338 (Paratyphi B-III); Gene 11 = SEECH997_RS20295 (Chester); Gene 12 = LFZ15_01345 (Hvittingfoss-I&II); Gene 13 = LFZ15_20305 (Hvittingfoss-III); Gene 14 = L098_RS21065 (Muenchen-I); Gene 15 = Muenchen-II. See Supplementary Table S2 for gene details. The potential error rate of serovar-specific genes was defined by the formula: (Number of FPs)^∗^(The frequency of that serovar in a given region)/(Total of genomes of that serovar). The specificity of typing rate was equal to (1 – potential error rate)*.

## Discussion

*Salmonella* serotyping has been vital for diagnosis and surveillance. Serovar prediction by traditional serotyping can be limited by the lack of surface antigen expression or autoagglutination properties (Wattiau et al., 2008). Recently, with the development of whole-genome sequencing technology, the relevant genomic regions of the *rfb* gene cluster for O antigen, gene *fliC* and gene *fljB* for H antigens, and genes targeted by MLST can be extracted and used for serovar identification. Several studies have identified serovar-specific genes or DNA fragments for serotyping through whole-genome sequencing based genomic comparison (Zou et al., 2013, 2016; Laing et al., 2017). However, these serovar-specific genes or DNA fragments only distinguished a small number of serovars. In this study, we identified 414 candidate serovar-specific or lineage-specific gene markers for 106 serovars which include 24 polyphyletic serovars and the paraphyletic serovar Enteritidis. A subset of these gene markers were validated by independent genomes and were able to assign serovars correctly in 95.3% of cases.

The above analysis was complicated by the presence of polyphyletic serovars, which arise independently from separate ancestors to form separate lineages. Therefore, a combination of lineage-specific gene markers was required for the clear identification of the majority of the polyphyletic serovars. Interestingly four polyphyletic serovars, Bredeney, Kottbus, Livingstone, and Virchow, each had one candidate serovar-specific gene marker which was present in all isolates of that serovar. The Bredeney serovar-specific gene was predicted to encode a translocase involved in O antigen conversion and could have been gained in parallel. The serovar-specific genes of the other three polyphyletic serovars encode hypothetical proteins with unknown function and no apparent explanation for their presence in different lineages of the same serovar.

Unlike polyphyletic serovars, the three lineages (clade A, B, and C) of the paraphyletic serovar Enteritidis share a recent common ancestor. Clade A and C are ancestral to Clade B. Previous studies described that Enteritidis was clustered with serovars Dublin, Berta, and Gallinarium which was called “Section Enteritidis” (Vernikos et al., 2007; Achtman et al., 2012; Allard et al., 2013; Timme et al., 2013). Another study showed that serovar Nitra was embedded within Enteritidis lineages by using whole genome phylogeny (Deng et al., 2014). There also was cross-reactivity between Enteritidis and Nitra according to Ogunremi’s study (Ogunremi et al., 2017). In our study, we selected the isolates based on rSTs, Nitra was not present in Enterobase rMLST database when this study commenced and so was not included in this study. Gallinarium is distinguishable from Enteritidis using the presence of a 4 bp deletion in the *speC* gene (Kang et al., 2011). We observed that the common ancestors of serovars Dublin, Berta, and Gallinarium, arose from an ancestor between Clades B and A/C. While Dublin can be separately identified, we cannot distinguish Berta or Gallinarium from Enteritidis clade A/C. These results highlight a limitation of the approach as serovars must be sufficiently divergent that they differ by at least one unique gene. Similarly, there were 8 other serovars that were not distinguishable likely due to very recent shared ancestry with little gene acquisition.

Serovar-specific candidate gene markers or lineage-specific candidate gene markers in 69 out of 106 serovars were contiguous in the genome with similar functions grouped together (data not shown). This suggests that these gene markers may have been incorporated into serovar genomes together through horizontal gene transfer. Indeed the seven Typhimurium specific candidate gene markers identified in this study (STM4492, STM4493, STM4494, STM4495, STM4496, STM4497, and STM4498) were located in Typhimurium tRNA^leuX^ integrating conjugative element-related region including genes from STM4488 to STM4498, which is a known horizontal gene transfer hotspot (Bishop et al., 2005). Similarly five Enteritidis specific candidate gene markers identified (SEN1379, SEN1380, SEN1382, SEN1383, and SEN1383) were located in the Sdr I region (Agron et al., 2001) and the prophage-like GEI/φSE14 region (Santiviago et al., 2010). Both of these regions are linked to prophages, which suggests that these regions integrated into the genome of a common ancestor of the global Enteritidis clade and were derived from horizontal gene transfer.

Other methods for *in silico* serovar prediction are implemented in SeqSero (Zhang et al., 2015) and SISTR (Yoshida et al., 2016). Both of these methods examine genomic regions responsible for surface antigens while SISTR also implements a cgMLST scheme to examine overall genetic relatedness. Additionally, traditional 7 gene MLST and eBURST groups derived from it can also be used for *in silico* serovar determination (Achtman et al., 2012; Ashton et al., 2016; Robertson et al., 2018). Both SISTR and SeqSero provide higher discriminatory power than traditional serovar identification (Yachison et al., 2017). However, they have a number of drawbacks such as indistinguishable serovars having the same antigenic formula or antigenic determinants not being expressed (Robertson et al., 2018). In the current study, we examined *in silico* serovar prediction by screening genomes against a set of 131 serovar-specific gene markers. The approach provided serovar prediction by yielding “presence or absence” of individual serovar-specific gene marker or combination of gene markers in a query isolate. We show that serovar-specific gene markers have comparable accuracy to other *in silico* serotyping methods with 91.5% isolates from initial identification dataset and 84.8% isolates from a validation dataset assigned to the correct serovar (with no FN and FP). 10.5% of isolates from validation dataset can be assigned to a small subset of serovars containing the correct serovar (with varied FP). The specificity for *in silico* serovar prediction approach by serovar-specific gene markers was 95.3%, slightly higher than SISTR (95%) and SeqSero (82.8%) in the same dataset we tested. This result was similar to the specificities of SISTR and SeqSero reported by Yachison et al. (2017) which were 94.8 and 88.2%, respectively.

Our serovar-specific gene marker based method does not require the accurate examination of O antigen gene clusters or sequence variation of the H antigen genes which can be problematic. Our method also alleviates the need for the entire gene or genome sequence be assembled which is necessary in MLST or cgMLST based methods. Therefore, this approach may be useful for cases where very little sequence is available such as in metagenomics or culture free typing as well as providing a third alternative to confirm other analyses.

The identification of a set of gene markers able to uniquely identify all prevalent serovars in a region may also be useful in the development molecular assays. These assays would be useful in serotyping isolates where cultures are no longer obtained and traditional serotyping is therefore impossible. For example, a set of PCR assays could be designed that would allow the sensitive detection of specific gene markers, and therefore allow prediction of the serovar, from a clinical sample. Additionally, by eliminating the need to detect serovars that are very rarely observed in a region the number of these gene markers required to detect all major serovars in a region can be significantly reduced allowing for a more cost-effective assay.

## Conclusion

In this study we identified candidate serovar-specific gene markers and candidate lineage-specific gene markers for 106 serovars by characterizing the accessory genomes of a representative selection of 2258 strains as potential markers for *in silico* serotyping. We account for polyphyletic and paraphyletic serovars to provide a new method, using the presence or absence of these gene markers, to predict the serovar of an isolate from genomic data. The gene markers identified here may also be used to develop serotyping assays in the absence of an isolated strain which will be useful as diagnosis moves to culture independent and metagenomic methods.

## Author Contributions

MP and RL designed the study and provided critical revision of the manuscript. XZ and MP performed the bioinformatic analysis. XZ, MP, and RL analyzed the results. XZ drafted the manuscript.

## Conflict of Interest Statement

The authors declare that the research was conducted in the absence of any commercial or financial relationships that could be construed as a potential conflict of interest.

## Footnotes

1 https://www.ebi.ac.uk/ena

2 http://bioinf.spbau.ru/spades

3 http://bioinf.spbau.ru/quast

4 http://github.com/marbl/harvest

5 https://www.ncbi.nlm.nih.gov/

6 http://rast.theseed.org/FIG/rast.cgi

## Supplementary Material

FIGURE S1 The SNP based phylogenetic tree constructed by ParSNP showing the evolutionary relationships within and between serovars using 1344 representative isolates including 1258 isolates from 107 serovars examined in the study and 86 isolates from serovars with less than 5 rSTs which were otherwise excluded from the study.

TABLE S1 The final data set of 2258 high quality and consistent serovar prediction genomes representing 107 serovars.

TABLE S2 A total of 414 candidate serovar-specific genes including 295 serovar-specific genes and 119 lineage-specific genes.

TABLE S3 An additional 1089 validation isolates with serovar prediction results by SISTR, SeqSero and serovar-specific gene markers.

TABLE S4 A minimum of 131 genes for identification of 106 serovars.

TABLE S5 A set of 65 genes for identification of 46 common serovars.

DATA S1 Sequences of 131 serovar-specific gene markers.

## Abbreviations

FN: false negatives
FP: false positives
FPR: false positive rate
MLST: multi-locus sequence typing
NEPSS: National Enteric Pathogens Surveillance Scheme
PPV: positive predictive value
rSTs: ribosomal MLST STs
SISTR: *Salmonella in silico* typing resource
TN: true negatives
TNR: true negative rate
TP: true positives
TPR: true positive rate
