# Supplementary material for: In silico Identification of Serovar-Specific Genes for Salmonella Serotyping
Source: Front Microbiol. 2019 Apr 24;10:835. doi: 10.3389/fmicb.2019.00835 (PMC6491675; doi:10.3389/fmicb.2019.00835)

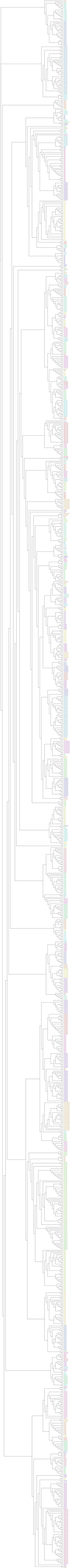

Supplement: FIGURE S1 — The SNP based phylogenetic tree constructed by ParSNP showing the evolutionary relationships within and between serovars using 1344 representative isolates including 1258 isolates from 107 serovars examined in the study and 86 isolates from serovars with less than 5 rSTs which were otherwise excluded from the study. [file Data_Sheet_1.PDF]
